# Association of Osteoarthritis With Changes in Structural Neuroimaging Markers Over Time Among Non-demented Older Adults

**DOI:** 10.3389/fnagi.2021.664443

**Published:** 2021-08-10

**Authors:** Lichuang Wu, Xiang Wang, Yiheng Ye, Cailong Liu

**Affiliations:** ^1^Department of Orthopaedics, The First Affiliated Hospital of Wenzhou Medical University, Wenzhou, China; ^2^Department of Orthopaedics, The First People’s Hospital of Longwan District, Wenzhou, China

**Keywords:** osteoarthritis, MRI, neuroimaging, dementia, longitudinal study

## Abstract

**Objective**: Although emerging evidence suggests that both osteoarthritis (OA) and brain atrophy (as assessed by structural neuroimaging markers) are associated with the risk of dementia, little is known about the association between OA and structural neuroimaging markers. This study aimed to examine the association of OA with changes in structural neuroimaging markers among non-demented older people.

**Methods**: We examined the cross-sectional and longitudinal associations between OA and structural neuroimaging markers (hippocampal volume, entorhinal volume, ventricular volume, and volume of gray matter of the whole brain) among non-demented older people. We categorized our participants as those without OA (OA−) and those with OA (OA+). At baseline, we included 1,281 non-demented older adults, including 1,050 without OA and 231 with OA.

**Results**: In the cross-sectional analysis, we did not observe any significant difference in structural neuroimaging markers between the two OA groups. In the longitudinal analysis, we found that compared to participants without OA, those with OA showed a steeper decline in volumes of the gray matter of the whole brain among non-demented older adults.

**Conclusions**: OA was associated with a steeper decline in volumes of the gray matter of the whole brain over time among non-demented older people.

## Introduction

Osteoarthritis (OA) is a common and debilitating joint disorder that contributes to functional impairment among older people (Hunter and Bierma-Zeinstra, 2019). The precise pathogenesis of OA remains largely unknown, though inflammation is thought to play an important role in the progression of OA (Hunter and Bierma-Zeinstra, 2019). For example, substantial evidence suggests that proinflammatory factors are involved in the development of disease (Staite et al., 1990; Ghivizzani et al., 1997; Lawlor et al., 2001). Interestingly, a previous retrospective population-based cohort study indicated that OA is also an independent risk factor for developing dementia (Huang et al., 2015). Further, a recent meta-analysis supported a link between OA and an increased risk of dementia (Weber et al., 2019). However, the mechanism through which OA increases the risk of dementia and cognitive impairment remains unclear.

Cerebral atrophy assessed on structural MRI is a valid marker of Alzheimer’s disease (AD) dementia (Mckhann et al., 2011). In addition, rates of change in several brain regions, such as entorhinal cortex (Cardenas et al., 2011), hippocampus (Jack et al., 2004), ventricular enlargement (Ridha et al., 2008), and whole-brain (Sluimer et al., 2010), have been reported to be associated with changes in cognitive performance, further suggesting that these brain regions are valid markers of disease progression. However, no previous studies have attempted to examine the association of OA with changes in these structural neuroimaging markers.

In the current study, at baseline, we examined the cross-sectional relationships between OA and several structural neuroimaging markers among non-demented older adults. Further, linear mixed models were conducted to examine the association of OA with changes in structural neuroimaging markers over time among non-demented older adults.

## Materials and Methods

### Alzheimer’s Disease Neuroimaging Initiative (ADNI) Database

Data utilized in the present study were extracted from the ADNI database^1^. The ADNI study has been described in detail elsewhere (Weiner et al., 2015). In brief, the ADNI aims to examine the progression of cognitive decline among MCI and mild AD patients using a variety of variables, including demographics, cognitive assessments, neuroimaging variables, and fluid biomarkers. In the present study, we included 1,281 non-demented older adults at baseline. We further categorized our participants into two groups based on their OA status: OA− group (*n* = 1,050) and OA+ group (*n* = 231). The OA status was reported by participants based on their medical history. At ADNI sites, local institutional review boards approved the study procedures, and participants provided written informed consent.

### Participants

In total, we included 1,281 non-demented older adults, including 415 individuals with normal cognition and 866 individuals with mild cognitive impairment (MCI). Individuals with normal cognition had a Clinical Dementia Rating (CDR; Morris, 1993) score of 0 and a Mini-Mental State Examination (MMSE; Folstein et al., 1975) score between 24 and 30. Individuals with MCI had a CDR score of 0.5, an MMSE score between 24 and 30, an objective memory impairment as evaluated by delayed recall scores of the Wechsler Memory Scale Logical Memory II, and an absence of clinical dementia. All the available data were used in this study.

### Cognitive Outcome

The global cognition of participants was assessed by the 13-item Alzheimer’s Disease Assessment Scale-Cognitive subscale (ADAS-Cog 13; Mohs et al., 1997).

### Structural MRI

The structural MRI data were extracted from the ADNI database. The procedure of neuroimaging analysis has been described in detail elsewhere (Jack et al., 2010). Four structural MRI markers were included in our analysis: hippocampal volume, entorhinal volume, ventricular volume, and volume of the gray matter of the whole brain. To adjust for the sex difference in head size (Sundermann et al., 2016), several transformations were made:

• Hippocampal volume ratio (HVR) = hippocampal/intracranial volume × 10^3^.
• Entorhinal volume ratio (EVR) = entorhinal/intracranial volume × 10^3^.
• Ventricular volume ratio (VVR) = ventricles/intracranial volume × 10^3^.
• Whole brain volume ratio (WVR) = whole brain/intracranial volume × 10^3^.
• In the current analysis, the neuroimaging data were extracted from the file “ADNIMERGE” on the ADNI website.

### Statistical Analysis

*T*-tests and *x*^2^ tests were used to examine differences in demographical variables and clinical variables between the two OA groups (OA− vs. OA+). Specifically, for continuous variables (age, education, ADAS-Cog 13, HVR, EVR, VVR, and WVR), t-tests were used to compare the difference between two groups, while *x*^2^ tests were used to compare the categorical variables between two groups (gender, APOE4 genotype, and MCI diagnosis). In addition, to examine the association of the OA status at baseline with changes in cognition and MRI markers over time, linear mixed models were conducted. All models included OA status, age, APOE4 genotype, gender, education, as well as their interactions with time. Every model also included a random intercept for each subject. Q-Q plots of residuals of our linear mixed models did not suggest any strong deviations from normality. All statistical work was conducted using *R* statistical software (version: 4.0.0; Team, 2013). The significance level was set at *p* < 0.05.

## Results

### Demographics and Clinical Characteristics by the OA Status

At baseline, we included 1,281 non-demented older adults, including 1,050 without OA and 231 with OA. As displayed in Table 1, we did not find any significant differences in demographics and clinical variables (age, gender, education, APOE4 genotype, MCI diagnosis, ADAS-Cog 13, HVR, EVR, VVR or WVR, all *p* > 0.05). There were missing values in our variables (HVR, EVR, VVR, and WVR) due to the quality or availability of MRI scans, resulting in varying numbers of participants used in our subsequent analyses (Table 1).

**Table 1:** Demographics and clinical characteristics by OA status.

Characteristics	OA−	OA+	*P* values
Age, years	73.5 ± 7.13	74.3 ± 6.79	0.07
Female gender, *n* (%)	446 (42.5)	113 (48.9)	0.07
Education, years	16 ± 2.82	16 ± 2.76	0.95
APOE4 carriers, *n* (%)	442 (42.1)	107 (46.3)	0.24
MCI diagnosis, *n* (%)	721 (68.7)	145 (62.8)	0.08
ADAS-Cog 13	14.3 ± 6.93	13.8 ± 7.21	0.33
HVR^a^	4.57 ± 0.775	4.64 ± 0.780	0.29
WVR^b^	673 ± 51	676 ± 50	0.33
VVR^c^	24.7 ± 12.7	24.8 ± 12.2	0.94
EVR^d^	2.36 ± 0.47	2.4 ± 0.49	0.24

*Abbreviations: OA, Osteoarthritis; MCI, Mild cognitive impairment; ADAS-Cog 13, the 13-item Alzheimer’s Disease Assessment Scale-Cognitive subscale; HVR, Hippocampal volume ratio; WVR, Whole brain volume ratio; VVR, Ventricles volume ratio; EVR, Entorhinal volume ratio. Notes. ^a^Included in the present analysis are 1,110 participants, including 918 in OA− group and 192 in OA+ group. ^b^Included in the present analysis are 1,259 participants, including 1,035 in OA− group and 224 in OA+ group. ^c^Included in the present analysis are 1,237 participants, including 1,017 in OA− group and 220 in OA+ group. ^d^Included in the present analysis are 1,112 participants, including 918 in OA− group and 194 in OA+ group*.

### Association of OA Status With Changes in Cognition and Neuroimaging Markers Over Time Among Non-demented Older Adults

**Figure 1:**
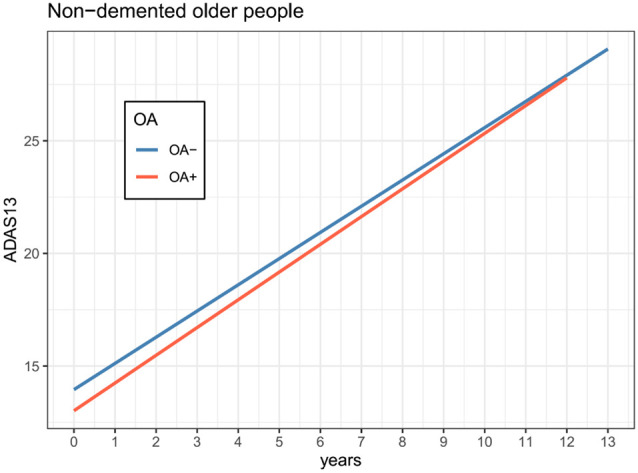
Association of OA status with changes in cognition over time among non-demented older adults. The OA status was not associated with the change in ADAS-Cog 13 over time among non-demented older adults (coefficient = −0.13, *p* = 0.08). Abbreviations: OA, Osteoarthritis; ADAS-Cog 13, the 13-item Alzheimer’s Disease Assessment Scale-Cognitive subscale.

**Figure 2:**
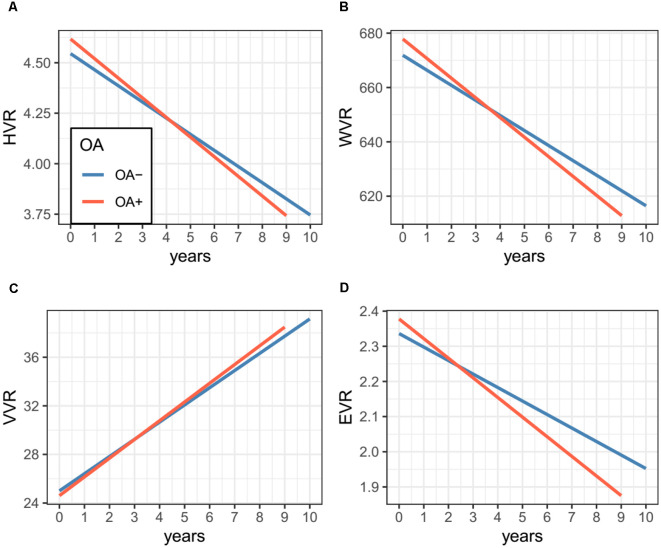
Association of OA status with changes in MRI markers over time among non-demented older adults. Panel **(A)** shows that OA status was not associated with the change in HVR. Panel **(B)** shows that compared with participants without OA, those with OA showed a significantly steeper decline in WVR. Panel **(C)** shows that OA status was not associated with a change in VVR. Panel **(D)** shows that OA status was not associated with the change in EVR. Abbreviations: OA, Osteoarthritis; HVR, Hippocampal volume ratio; WVR, Whole brain volume ratio; VVR, Ventricles volume ratio; EVR, Entorhinal volume ratio.

Results of linear mixed models are presented in Table 2. The association of OA status with change in ADAS-Cog 13 was marginally significant (Coefficient = −0.13, *p* = 0.08; Table 1 and Figure 1). However, as shown in Figure 2 and Table 2, we found that the OA status was significantly associated with change in WVR (Coefficient = −1.24, *p* < 0.001), but not HVR (Coefficient = −0.005, *p* = 0.2) or VVR (Coefficient = 0.003, *p* = 0.94). The association of OA status with change in EVR was marginally significant (Coefficient = −0.01, *p* = 0.065). Specifically, the OA status × time interaction term for WVR was significant, suggesting that compared with participants without OA, those with OA showed a significantly steeper decline in WVR (Figure 2B). For Figures with both regression lines and data points, please see the Supplementary Figures 1, 2.

**Table 2:** Association of OA status with changes in ADAS-Cog 13 and neurodegenerative markers over time among non-demented older people.

Predictors	Coefficient	SE	*P* values
		Outcome: ADAS-Cog 13
OA × time	−0.13	0.07	0.08
		Outcome: HVR
OA × time	−0.005	0.004	0.2
		Outcome: WVR
OA × time	−1.24	0.29	<0.0001
		Outcome: VVR
OA × time	0.003	0.05	0.94
		Outcome: EVR
OA × time	−0.01	0.01	0.065

*Abbreviations: OA, Osteoarthritis; ADAS-Cog 13, the 13-item Alzheimer’s Disease Assessment Scale-Cognitive subscale; HVR, Hippocampal volume ratio; WVR, Whole brain volume ratio; VVR, Ventricles volume ratio; EVR, Entorhinal volume ratio. Notes: All models were adjusted for age, education, gender, APOE4 genotype, and their interactions with time. However, coefficients of the main effects of these variables were not shown. A comprehensive list of regression terms can be found in Supplementary Tables 1–5*.

### Supplementary Analysis

In the present study, the dropout rate was high after the 4th year of follow up visit. To test the robustness of our results, we additionally conducted a supplementary analysis and restricted our analysis to those with follow-up duration ≤4 years. The association of OA status with change in WVR was still significant (Table 3).

**Table 3:** Association of OA status with change in WVR over time among non-demented older people with the duration of follow up ≦ 4 years.

Predictors	Coefficient	SE	*P* values
Time	−5.11	1.26	<0.001
OA	7.74	3.3	0.019
Age	−3.5	0.18	<0.001
Female gender	13	2.57	<0.001
APOE4 + genotype	−8.16	2.58	0.0015
Time × OA	−0.64	0.31	0.036
Time × Age	0.004	0.017	0.8
Time × Female gender	−0.49	0.23	0.035
Time × APOE4 + genotype	−2.2	0.23	<0.001

*Abbreviation: OA, Osteoarthritis*.

## Discussion

In the current study, we examined the cross-sectional and longitudinal association of the OA status with cognitive performance and several structural neuroimaging markers among non-demented older adults. In the cross-sectional analysis, no significant difference in ADAS-Cog 13 or structural neuroimaging markers was observed, suggesting that these outcome measures were similar between the two group at baseline. In the longitudinal analysis, linear mixed models showed that OA status was associated with the change in volumes of the gray matter of the whole brain, but not ADAS-Cog 13, hippocampal volume, entorhinal volume, or ventricular volume.

The rate of decline in volumes of the gray matter of the whole brain has been reported to be associated with cognitive decline, supporting its role as a valid marker of clinical progression (Fox et al., 1999; Josephs et al., 2008; Schott et al., 2008; Sluimer et al., 2008). However, no previous studies have attempted to examine the association of OA status with change in WVR over time among non-demented older people. The finding of the present study that OA was associated with the steeper decline in volumes of the gray matter of the whole brain among non-demented older adults is in line with the results of previous studies, which suggested a relationship between OA and the risk of developing dementia (Huang et al., 2015; Chen et al., 2018; Weber et al., 2019). In a population-based cohort study, Huang and colleagues have suggested that OA is an independent risk factor for developing dementia (Huang et al., 2015). Another study also indicated that compared to subjects without OA, those with OA are at a higher risk of dementia (Chen et al., 2018). Recently, a meta-analysis further supported an association between OA and the risk of developing dementia (Weber et al., 2019). However, no study has attempted to examine the association of OA status with change in structural neuroimaging markers among non-demented older adults. To our knowledge, this is the first study to examine the longitudinal association between OA and several structural neuroimaging markers (hippocampal volumes, entorhinal volumes, ventricular volumes, and volumes of gray matter of the whole brain) among non-demented older adults.

The precise mechanisms underlying this association remain unknown, though there are several possible mechanisms that may accelerate the reduction of brain volumes in subjects with OA. First, it is possible that peripheral inflammatory cytokines induced by OA may trigger neuroinflammation, which leads to the accumulation of AD pathologies and neurodegeneration. For instance, in an animal study, Kyrkanides and colleagues suggested that induction of OA and peripheral inflammation contributes to the progression of neuroinflammation and the deposition of AD pathology (Kyrkanides et al., 2011). Further, systemic inflammation induced by LPS could lead to the release of inflammatory cytokines in the brain that promotes cognitive decline and neurodegeneration (Cunningham et al., 2005, 2009). Second, it is possible that the association of OA with changes in brain volumes may be mediated by other factors. For instance, patients with OA demonstrate a reduced level of physical activity, which has been reported to be associated with a higher risk of developing dementia and decline in volumes of several brain regions (Buchman et al., 2012; Blondell et al., 2014; Erickson et al., 2014).

Several potential limitations should be addressed. First, we categorized our participants into two OA groups (OA− vs. OA+) based on the self-report of OA history, which may lead to some misclassifications in both groups. In future studies, the diagnosis of OA should be based on standardized diagnostic criteria. Second, given the nature of our study design, we could not provide any evidence on causation according to our findings. Third, participants in the ADNI study represent a convenience sample, which may limit our ability to generalize our findings to other populations. Finally, the associations of OA with changes in ADAS-Cog 13 and EVR were marginally significant, indicating that there was a tendency towards an effect. Therefore, further studies are needed to replicate and validate our results.

In conclusion, OA was associated with a steeper decline in volumes of the gray matter of the whole brain over time among non-demented older people.

## Data Availability Statement

The datasets presented in this article are not readily available because the ADNI data utilized in the current study are publically accessible. For more infomation, please visit: adni.loni.usc.edu.

## Ethics Statement

At ADNI sites, local institutional review boards approved the study procedures, and participants provided written informed consent.

## Author Contributions

LW performed the statistical analysis, drafted this article, and interpreted the data. XW contributed to data collection, literature search, and statistical analysis. YY critically revised this article. CL was involved in the design of this study and supervised this project. All authors contributed to the article and approved the submitted version.

## Conflict of Interest

The authors declare that the research was conducted in the absence of any commercial or financial relationships that could be construed as a potential conflict of interest.

## Publisher’s Note

All claims expressed in this article are solely those of the authors and do not necessarily represent those of their affiliated organizations, or those of the publisher, the editors and the reviewers. Any product that may be evaluated in this article, or claim that may be made by its manufacturer, is not guaranteed or endorsed by the publisher.

## Footnotes

^1^ adni.loni.ucla.edu
