# Supplementary material for: Association of Osteoarthritis With Changes in Structural Neuroimaging Markers Over Time Among Non-demented Older Adults
Source: Front Aging Neurosci. 2021 Aug 10;13:664443. doi: 10.3389/fnagi.2021.664443 (PMC8383489; doi:10.3389/fnagi.2021.664443)
Supplement: Supplementary file 1 [file Data_Sheet_1.docx]

**Supplementary Table 1.** Association of OA status with change in ADAS-Cog 13 over time among non-demented older people

| Predictors | Coefficient | SE | P values |
| --- | --- | --- | --- |
| Time | -0.4 | 0.3 | 0.19 |
| OA | -1.0 | 0.64 | 0.11 |
| Age | 0.2 | 0.04 | < 0.0001 |
| Female gender | -1.47 | 0.5 | 0.003 |
| APOE4+ genotype | 4.0 | 0.5 | < 0.0001 |
| Time × OA | -0.13 | 0.07 | 0.08 |
| Time × Age | 0.015 | 0.004 | 0.0002 |
| Time × Female gender | 0.23 | 0.05 | < 0.0001 |
| Time × APOE4+ genotype | 1.1 | 0.06 | < 0.0001 |

Abbreviations: OA: Osteoarthritis; ADAS-Cog 13: the 13-item Alzheimer’s Disease Assessment Scale- Cognitive subscale.

**Supplementary Table 2.** Association of OA status with change in HVR over time among non-demented older people

| Predictors | Coefficient | SE | P values |
| --- | --- | --- | --- |
| Time | 0.03 | 0.016 | 0.08 |
| OA | 0.09 | 0.05 | 0.1 |
| Age | -0.04 | 0.003 | < 0.0001 |
| Female gender | 0.29 | 0.04 | < 0.0001 |
| APOE4+ genotype | -0.27 | 0.04 | < 0.0001 |
| Time × OA | -0.005 | 0.004 | 0.2 |
| Time × Age | -0.001 | 0.002 | < 0.0001 |
| Time × Female gender | -0.02 | 0.003 | < 0.0001 |
| Time × APOE4+ genotype | -0.06 | 0.003 | < 0.0001 |

Abbreviations: OA: Osteoarthritis; HVR: Hippocampal volume ratio.

**Supplementary Table 3.** Association of OA status with change in WVR over time among non-demented older people

| Predictors | Coefficient | SE | P values |
| --- | --- | --- | --- |
| Time | -4.6 | 1.16 | 0.0001 |
| OA | 8.3 | 3.3 | 0.012 |
| Age | -3.5 | 0.18 | < 0.0001 |
| Female gender | 13.2 | 2.58 | < 0.0001 |
| APOE4+ genotype | -7.96 | 2.58 | 0.002 |
| Time × OA | -1.24 | 0.29 | < 0.0001 |
| Time × Age | 0.004 | 0.02 | 0.78 |
| Time × Female gender | -7.7 | 0.2 | 0.0001 |
| Time × APOE4+ genotype | -2.45 | 0.21 | < 0.0001 |

Abbreviations: OA: Osteoarthritis; WVR: Whole brain volume ratio.

**Supplementary Table 4.** Association of OA status with change in VVR over time among non-demented older people

| Predictors | Coefficient | SE | P values |
| --- | --- | --- | --- |
| Time | 0.19 | 0.19 | 0.32 |
| OA | -0.6 | 0.89 | 0.49 |
| Age | 0.69 | 0.05 | < 0.0001 |
| Female gender | -5.2 | 0.69 | < 0.0001 |
| APOE4+ genotype | 0.8 | 0.69 | 0.2 |
| Time × OA | 0.003 | 0.05 | 0.94 |
| Time × Age | 0.01 | 0.003 | < 0.0001 |
| Time × Female gender | -0.06 | 0.03 | 0.06 |
| Time × APOE4+ genotype | 0.76 | 0.03 | < 0.0001 |

Abbreviations: OA: Osteoarthritis; VVR: Ventricles volume ratio.

**Supplementary Table 5.** Association of OA status with change in EVR over time among non-demented older people

| Predictors | Coefficient | SE | P values |
| --- | --- | --- | --- |
| Time | 0.03 | 0.02 | 0.17 |
| OA | 0.05 | 0.035 | 0.14 |
| Age | -0.02 | 0.002 | < 0.0001 |
| Female gender | 0.05 | 0.03 | 0.087 |
| APOE4+ genotype | -0.16 | 0.03 | < 0.0001 |
| Time × OA | -0.01 | 0.006 | 0.065 |
| Time × Age | -0.0007 | 0.0003 | 0.024 |
| Time × Female gender | -0.02 | 0.004 | 0.0001 |
| Time × APOE4+ genotype | -0.03 | 0.004 | < 0.0001 |

Abbreviations: OA: Osteoarthritis; EVR: Entorhinal volume ratio.


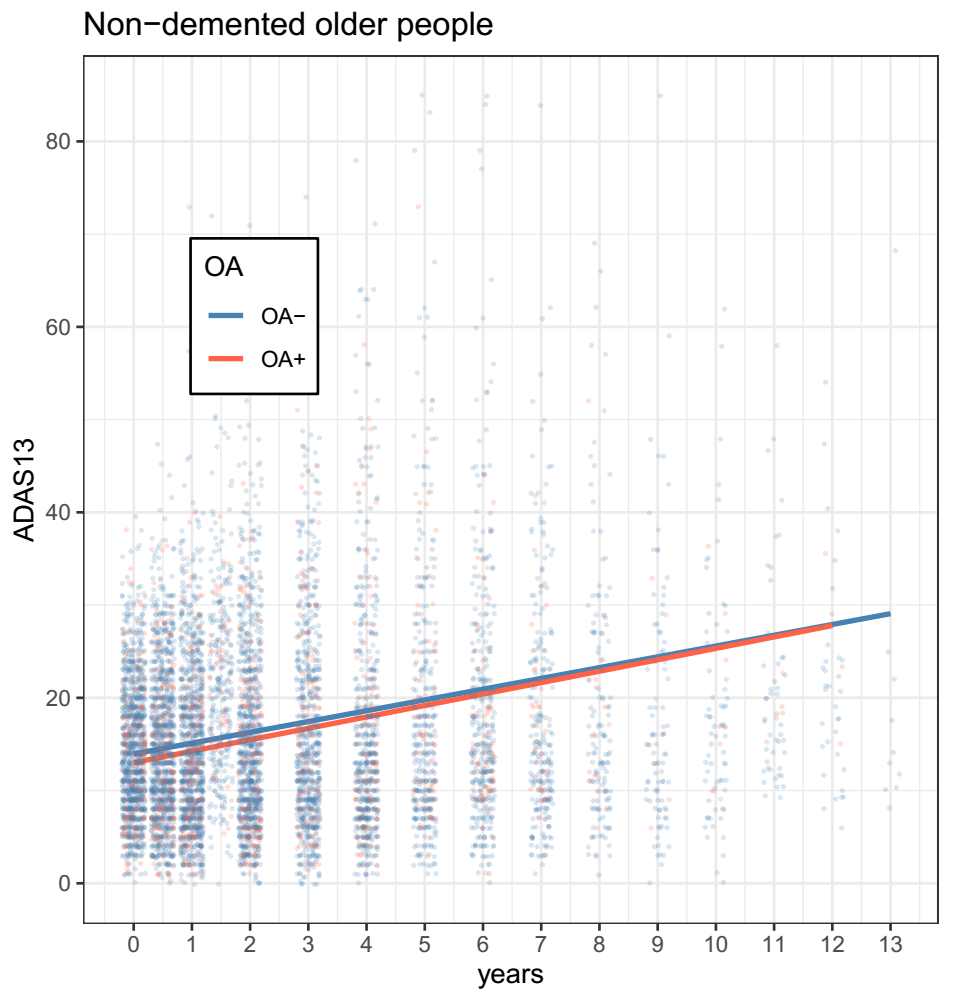


**Supplementary Figure 1**. Association of OA status with changes in cognition over time among non-demented older adults.


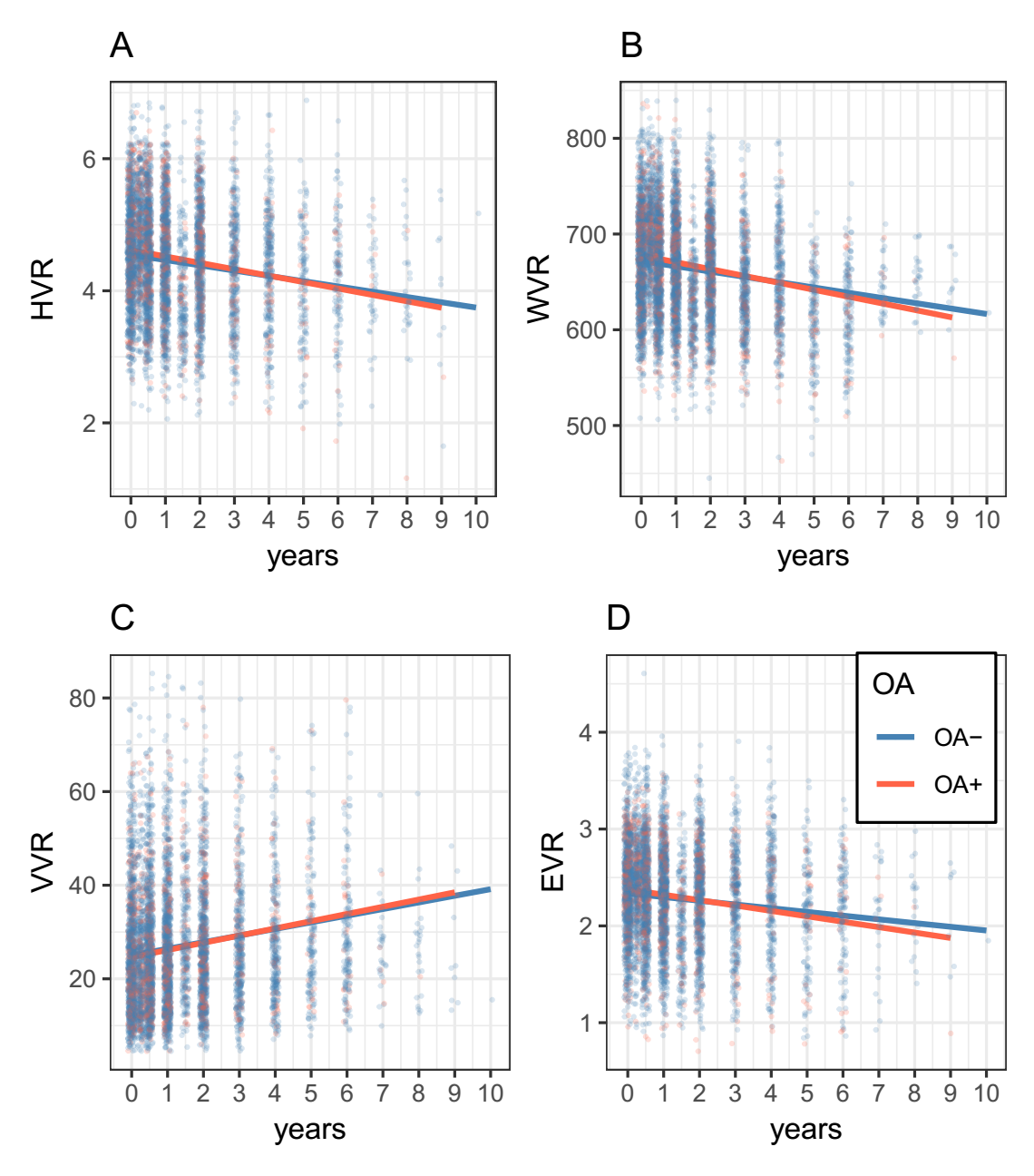


**Supplementary Figure 2**. Association of OA status with changes in MRI markers over time among non-demented older adults.
